# Metabolomic and Transcript Analysis Revealed a Sex-Specific Effect of Glyphosate in Zebrafish Liver

**DOI:** 10.3390/ijms23052724

**Published:** 2022-03-01

**Authors:** Christian Giommi, Claudia Ladisa, Oliana Carnevali, Francesca Maradonna, Hamid R. Habibi

**Affiliations:** 1 Dipartimento Scienze della Vita e dell’Ambiente, Università Politecnica delle Marche, Via Brecce Bianche, 60131 Ancona, Italy; c.giommi@pm.univpm.it (C.G.); o.carnevali@univpm.it (O.C.); 2 Department of Biological Sciences, University of Calgary, Calgary, AB T2N 1N4, Canada; claudia.ladisa1@ucalgary.ca (C.L.); habibi@ucalgary.ca (H.R.H.); 3 INBB—Consorzio Interuniversitario di Biosistemi e Biostrutture, 00136 Roma, Italy

**Keywords:** *Danio rerio*, metabolomic, liver, glyphosate, Roundup^®^, purine metabolism, oxidative stress

## Abstract

Glyphosate is a component of commonly used herbicides for controlling weeds in crops, gardens and municipal parks. There is increasing awareness that glyphosate-based herbicides, in addition to acting on plants, may also exert toxicity in wildlife and humans. In this study, male and female adult zebrafish were exposed to 700 µg/L of glyphosate (GLY), for 28 days. We used the metabolomic approach and UHPLC-ESI-MS to analyze liver samples to investigate the adverse effects of glyphosate on hepatic metabolism. The impact of GLY was found to be sex-specific. In female, GLY exposure affected purine metabolism by decreasing the levels of AMP, GMP and inosinic acid, consequently increasing uric acid levels with respect to the control (CTRL). Exposure to GLY also caused a decrease of UMP levels in the pyrimidine metabolism pathway. In male, GLY exposure decreased the aminoadipic acid within the lysine degradation pathway. Transcript analysis of genes involved in stress response, oxidative stress and the immune system were also performed. Results demonstrated an increased stress response in both sexes, as suggested by higher *nr3c1* expression. However, the *hsp70.2* transcript level was increased in female but decreased in male. The results demonstrated reduced *sod1*, *sod2*, and *gpx1a* in male following exposure to GLY, indicating an impaired oxidative stress response. At the same time, an increase in the *cat* transcript level in female was observed. mRNA levels of the pro-inflammatory interleukins *litaf* and *cxcl8b.1* were increased in female. Taken together, the results provide evidence of disrupted nucleotide hepatic metabolism, increased stress inflammatory response in female and disruption of oxidative stress response in male.

## 1. Introduction

In both plants and microorganisms, the mechanism of action of the non-selective herbicide glyphosate (GLY), which is an amino phosphonic analogue of the amino acid glycine, relies on the inhibition of the enzyme 5-enolpyruvylshikimate-3-phosphate synthase (EPSPS) activity, leading to reduced aromatic amino acid synthesis [1]. To improve absorption, glyphosate isopropylamine salt is used in combination with polyethoxylated tallow amine (POEA) which is the most utilized form of coadjutants. These formulations are sold under the trade name of Roundup^®^, in which the active ingredient is indicated as glyphosate acid equivalent (a.e.) and could be present at different concentrations. Currently, this herbicide is used extensively to eliminate weeds in conjunction with the use of genetically modified GLY-resistant plants. These formulations are also found in municipal parks as well as public and private gardens. There is evidence that this compound can reach surface waters via direct applications and runoffs. Limits for the presence of GLY in drinking water range from the highest levels in Australia (1000 µg/L) and USA (700 µg/L) to lower levels in Canada (280 µg/L) and Europe (0.1 µg/L) [2]. Despite the claim that GLY-based herbicides only target plant-specific enzymatic pathway, various studies demonstrated adverse effects of this contaminant in both vertebrate and invertebrates [3,4,5], as well as in wildlife and aquatic species [6,7,8,9,10], where this contaminant, alone or in formulation, induced oxidative stress and DNA damage. These observations are raising concern about public health since GLY can be detected in the urine of humans [11]. Thus we used a metabolomic- and transcriptomic-based approach to investigate the toxicity of GLY alone and the equivalent concentration of GLY in combination with coadjutants present in Roundup^®^ in zebrafish.

## 2. Results

### 2.1. Metabolomic Characterization of the Liver

**Figure 1:**
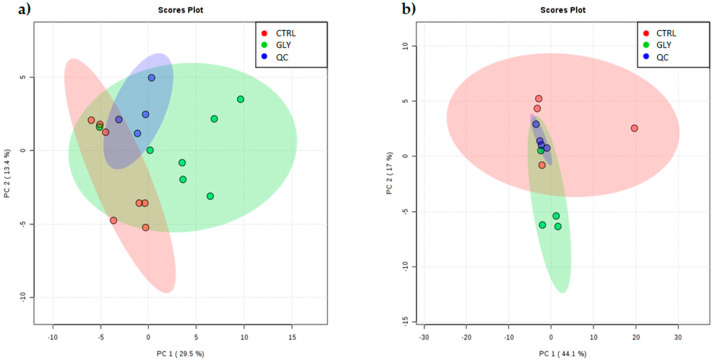
PCA score plot of CTRL and GLY metabolic profile in female and male livers. PCA score scatter plot of CTRL (red) vs. GLY (green) and QC (blue) in (**a**) female and (**b**) male livers. The abscissa axis shows the PC1 while the ordinate axis shows the PC2; the percentage of total variance for each PC is shown in parentheses. All samples are comprised within the 95% confidence interval of their respective group showing the absence of outliers. QC samples show strong cluster formation.

A metabolomic approach was used to analyze GLY-induced changes of hepatic metabolism. Data generated from ultra-high-performance liquid chromatography coupled with high-resolution full-scan mass spectroscopy (UHPLC-MS) were visualized on MAVEN software as peaks with intensity corresponding to the concentration of metabolites in the samples. MAVEN, together with a standard library of mass to charge (m/z) and retention times of metabolites, allowed the identification of 71 metabolites in zebrafish liver extracts, with levels differing between CTRL and GLY groups. The metabolic profile was studied by means of multivariate analysis and visualization techniques, including principal component analysis (PCA) and partial-least squares-discriminant analysis (PLS-DA). Seven extracted pools of female livers and four extracted pools of male livers were used to perform PCA with the quality control group (QC, *n* = 4), to investigate the presence of outliers and assess the dataset reliability. In this regard, the PCA scatter plot demonstrated a strong cluster formation for the QC group (Figure 1a,b) in females and males, despite lacking a clear separation between the groups.

**Figure 2:**
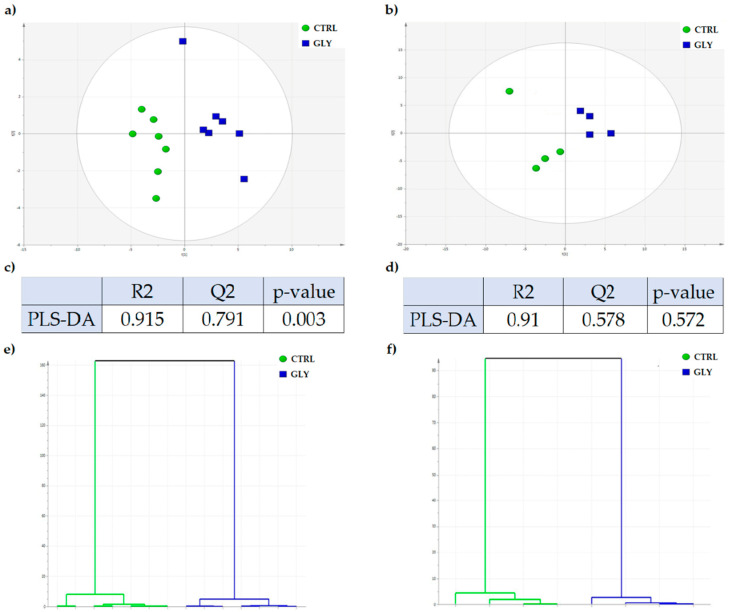
PLS-DA score plot and hierarchical analysis in CTRL and GLY females and males. PLS-DA score scatter plot of CTRL (green) vs. GLY (blue) in (**a**) females and (**b**) males. (**c**,**d**) Quality parameters (R2 and Q2) and *p*-value of the two PLS-DA-built models in females and males, respectively. Hierarchical analysis of CTRL (green) vs. GLY (blue) in (**e**) females and (**f**) males.

PLS-DA analysis was then performed to investigate differences between treatment groups in both male and female fish. The quality parameters (R2 and Q2) and *p*-values for the built models, were estimated for the analysis by the SIMCA program. Since the good quality of a built model is set for R2 and Q2 > 0.5, it can be assumed that a good and significant separation was observed between the CTRL and GLY-exposed groups in females (*p* < 0.003; R2 = 0.915, Q2 = 0.791) (Figure 2a,c). However, in males, the cluster separation was not significantly different between the CTRL and GLY-exposed groups (*p* < 0.572; R2 = 0.91, Q2 = 0.578) despite the good quality of the model (Figure 2b,d). Hierarchical analysis revealed separation of treatment groups in both sexes (Figure 2e,f).

The variable importance in projection (VIP) score was used to determine the importance of metabolites in the built models. A VIP score ≥ 1 is usually considered as threshold for the selection of individual metabolites used for the SIMCA PLS-DA model building and are then used for further analysis (Table 1a,b).

### 2.2. Univariate Analysis to Identify Metabolic Profiles

**Figure 3:**
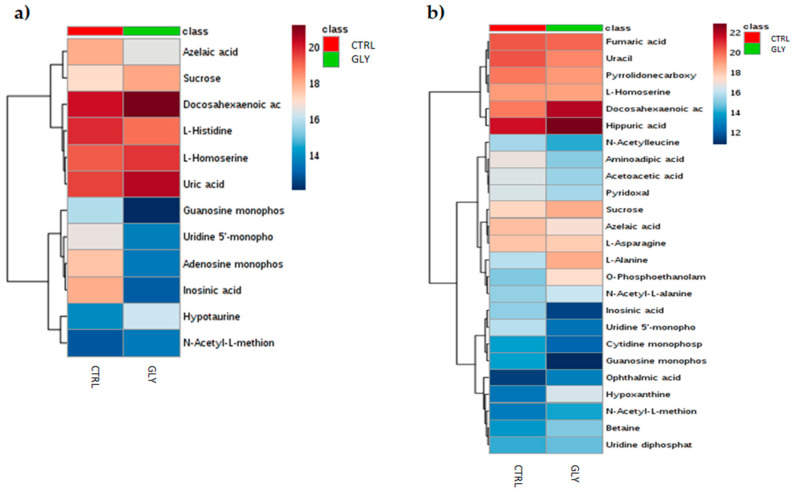
Clustered heatmap of VIP > 1 in CTRL and GLY-exposed female and male livers. Heatmap showing the VIP > 1 metabolites found in multivariate analysis (PLS-DA) of CTRL vs. GLY in (**a**) females and (**b**) males. Euclidean distance was used to perform clustering analysis. The heatmaps were constructed using Ward’s algorithm and samples are shown as group average. Increase and decrease in metabolite concentration are indicated by red and blue shades, respectively; the more intense is the color, the greater the increase or the decrease.

Those metabolites that following the PLS-DA model development, resulted were significant were used to perform univariate analysis on the MetaboAnalyst 5.0 online platform. In order to investigate changes at metabolite level (FDR < 0.05), multiple t-tests were performed on CTRL and GLY-exposed groups using VIP > 1 metabolites (GLY female = 12; GLY male = 25) in both male and female. To summarize the results, heatmaps were also provided, as shown in Figure 3a,b.

In female, exposure to GLY significantly increased sucrose, hypotaurine, L-homoserine, uric acid (UA), and docosahexaenoic acid (DHA), but decreased azelaic acid, adenosine monophosphate (AMP), uridine-5′-monophosphate (UMP), L-histidine (His), guanosine monophosphate (GMP), and inosinic acid (IMP) (Table 2a).

**Figure 4:**
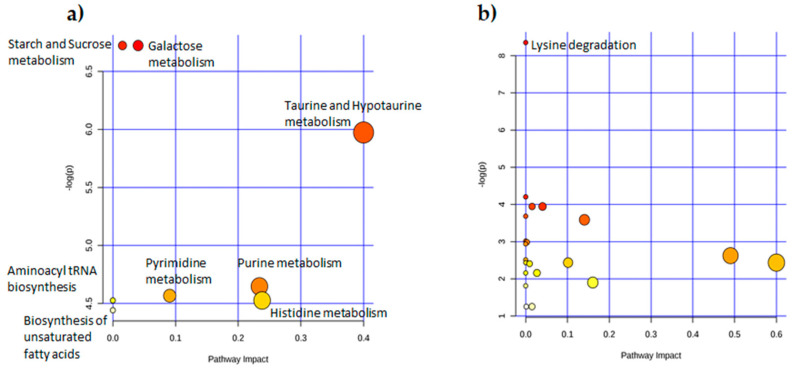
Dot plot of GLY-modulated pathways in the liver. MetPA graph showing main altered metabolic pathways found in CTRL vs. GLY comparison in (**a**) females and (**b**) males. The color of the dot (from white to red) reflects the significance (FDR < 0.05) of the pathway. The dimension of the dots reflects the impact of the metabolites in that pathway, the bigger the dot, the greater the impact of the metabolites in the pathway. The names of the pathways that were statistically significantly altered (FDR < 0.05) are reported.

Metabolomic pathway analysis (MetPA) is able to trace the involvment of metabolites within metabolic pathways. The analysis was performed using MetaboAnalyst 5.0 starting from the same dataset used for the multiple t-test. Sucrose was found to be involved in galactose, starch, and sucrose metabolism pathways. The AMP, IMP, GMP, and UA were involved in the purine metabolism pathway, while UMP was involved in the pyrimidine metabolism pathway. Hypotaurine was found in taurine and hypotaurine metabolism, DHA was found in the biosynthesis of unsaturated fatty acids and His in histidine and β-alanine metabolism as well as aminoacyl tRNA biosynthesis pathways (Table 3a and Figure 4a).

According to univariate analysis, only aminoadipic acid was significantly decreased in the GLY-exposed males (Table 2b), with MetPA identifying this metabolite inside the lysine degradation pathway (Table 3b and Figure 4b) which was the only pathway significantly affected.

### 2.3. Liver Transcript Analysis

**Figure 5:**
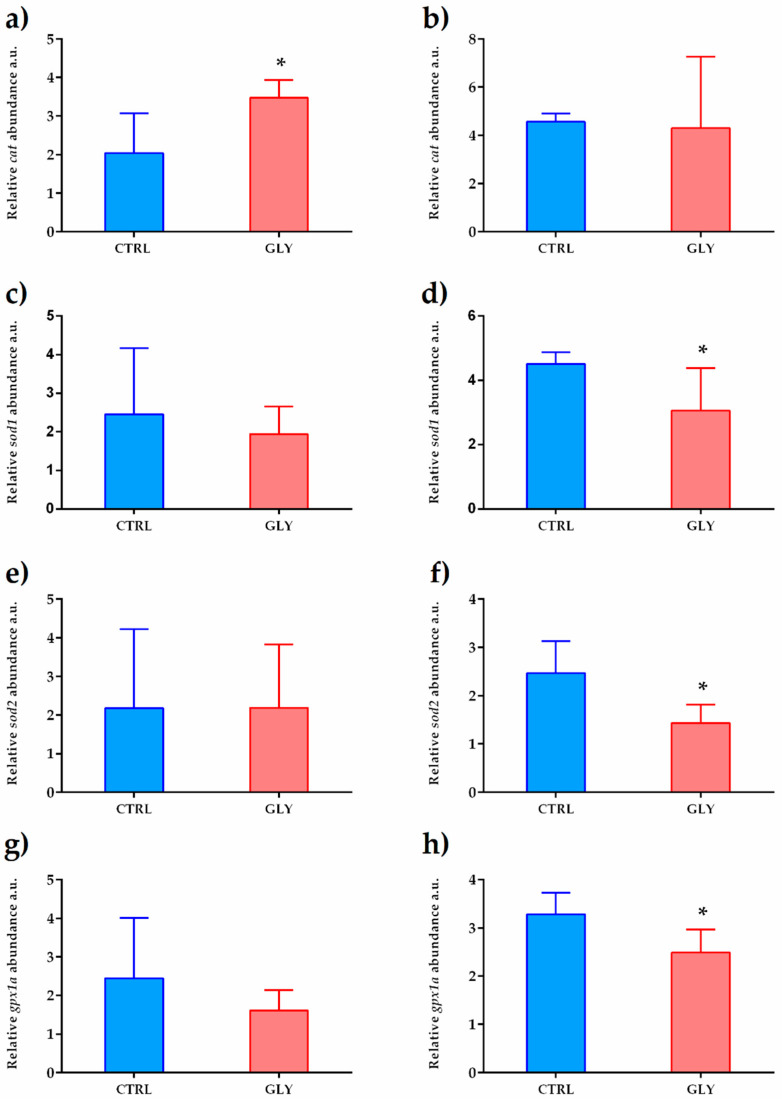
Gene expression profiles of hepatic oxidative stress response biomarkers. *cat* (**a**,**b**) *sod1* (**c**,**d**), *sod2* (**e**,**f**), and *gpx1a* (**g**,**h**) mRNA levels in female and male livers, respectively, normalized against *rplp0* and *rplp13* in CTRL and GLY-exposed zebrafish. Data are shown as mean ± SD (*n* = 5) and were analyzed by the t-test. Asterisks above each column denote significant differences between the experimental groups (* *p* < 0.05).

Based on transcript analysis, GLY exposure induced a different phenotype in females and males, while UA was upregulated in female livers. Considering that UA is able to induce oxidative stress when chronically elevated, genes involved in oxidative stress response were evaluated. Results clearly supported this hypothesis since GLY exposure significantly increased *cat* mRNA level in females, while in males, *sod1*, *sod2*, and *gpx1a* were reduced (Figure 5).

**Figure 6:**
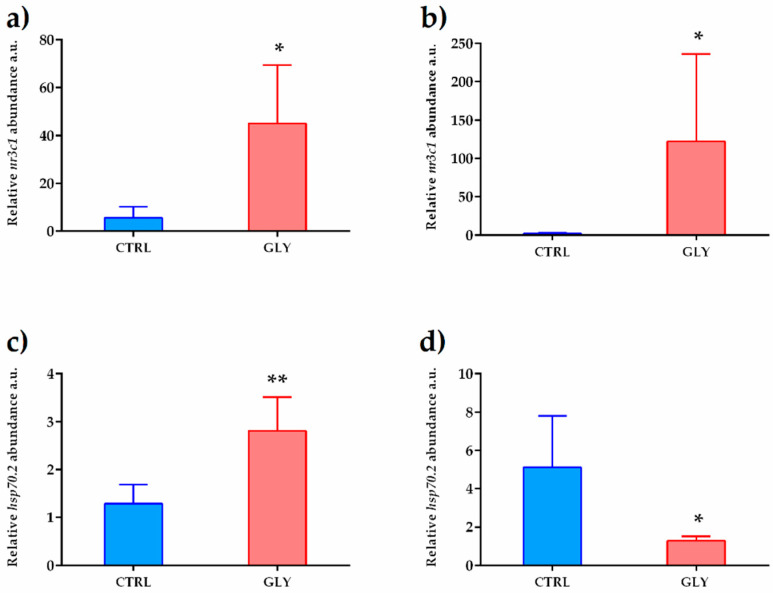
Gene-expression profile of stress-response biomarkers. *nr3c1* (**a**,**b**) and *hsp70.2* (**c**,**d**) mRNA levels in female and male livers, respectively, normalized against *rplp0* and *rplp13* in CTRL and GLY-exposed zebrafish. Data are shown as mean ± SD (*n* = 5) and were analyzed by the *t*-test. Asterisks above each column denote significant differences between the experimental groups (* *p* < 0.05, ** *p* < 0.01).

Furthermore, GLY exposure increased *nr3c1* levels in both sexes, while *hsp70.2* transcript level was increased in females and decreased in males (Figure 6). These two genes, as stress response genes, were reliable biomarkers of GLY exposure and their levels provided information on the organism’s oxidative-stress and immune-system responses.

**Figure 7:**
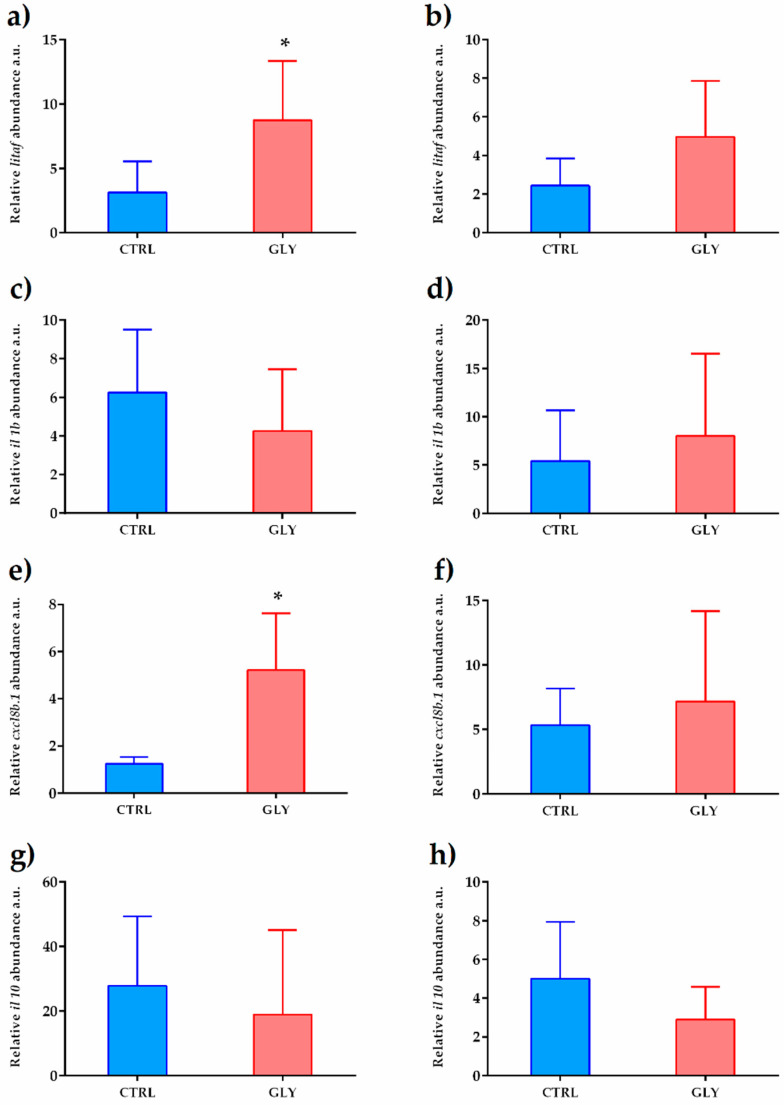
Gene expression profile of immune system biomarkers. *litaf* (**a**,**b**), *il1b* (**c**,**d**), *cxcl8b.1* (**e**,**f**), and *il10* (**g**,**h**) mRNA levels in female and male livers, respectively, normalized against *rplp0* and *rplp13* in CTRL and GLY-exposed zebrafish. Data are shown as mean ± SD (*n* = 5) and were analyzed by the *t*-test. Asterisks above each column denote significant differences between the experimental groups (* *p* < 0.05).

Since immune system and oxidative stress are related processes, a set of genes involved in the immune system response was evaluated. In males, no significant changes were found regarding those genes involved in immune response, while in females, levels of pro-inflammatory gene transcripts, interleukins *litaf* and *cxcl8b.1*, were increased (Figure 7).

**Figure 8:**
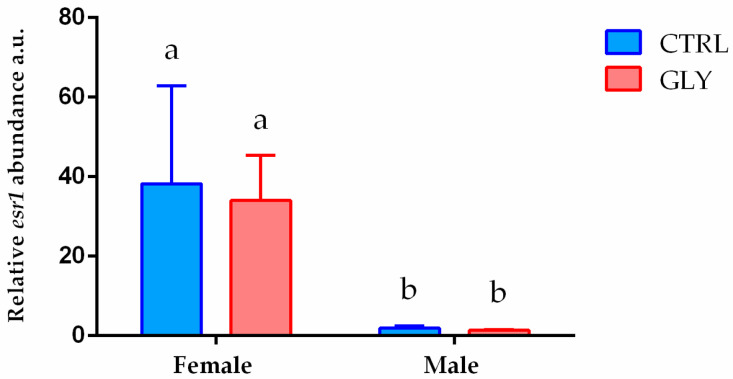
Gene expression profiles of hepatic *esr1. esr1* mRNA levels in female and male livers normalized against *rplp0* and *rplp13* in CTRL and GLY-exposed zebrafish. Data are shown as mean ± SD (*n* = 5) and were analyzed by two-way ANOVA followed by Tukey’s multiple comparison test. Letters above each column denote significant differences among the experimental groups (*p* < 0.05).

*Esr1* mRNA levels were further analyzed in female and male livers to compare differences in mRNA abundance between sexes. In females, *esr1* mRNA levels were significantly higher than in male fish. Treatment did not affect basal mRNA levels in either males or females (Figure 8).

## 3. Discussion

A metabolomic- and a transcriptomic-based approach was conducted on female and male zebrafish livers to investigate hepatic metabolic changes after chronic exposure to glyphosate. The initial experimental design also included one more group exposed to one dose of the commercial formulation Roundup^®^, containing an equivalent concentration of glyphosate (700 µg/L). However, all fish died one hour after the addition of the Roundup. Likely, GLY alone did not cause mortality, and other compounds in the Roundup^®^ made the mixture very toxic. Indeed, as previously demonstrated [12], the adverse effects of this herbicide on the aquatic ecosystem are attributable to the co-adjuvants.

Multivariate analysis of liver metabolism in females showed a significant shift in the GLY-treated fish compared to the CTRL fish. The univariate analysis revealed sex-specific differences in the GLY response, with females showing greater susceptibility to deleterious effects of the contaminant. In females, we observed disruption of purine metabolism, highlighted by reduction of purine intermediates AMP, IMP, and GMP, together with a decrease of the pyrimidine intermediate, UMP. Decrease of these metabolites could result from depletion of their precursor molecule, phosphoribosyl pyrophosphate (PRPP) [13]. The utilization of AMP, IMP, and GMP could lead to an increased level of UA which is the final product of the purine metabolism pathway, associated with increased oxidative stress [14]. Similar results were previously observed in goldfish, *Carassius auratus*, exposed to Nongteshi^®^ [15] which is an herbicide containing 30% (*w*/*v*) GLY, leading to decreased inosine and GTP.

In the present study, we observed depletion of His in the liver of GLY-exposed females. There is evidence that the essential amino acid His has antioxidant, anti-inflammatory, and metal-ion chelation properties when supplied through the diet [16]. The primary source of His is the diet and protein turnover, and its metabolism leads to the production of carnosine, homocarnosine, histidine-rich protein, histamine, and glutamic acid [16]. His depletion observed in the female liver could be a contributing factor for oxidative stress induced by the contaminant. Other studies in fish demonstrated that a low level of His increases the tolerance to hypoxia [17], leading to stress conditions and the formation of ROS. Furthermore, His supplementation was observed to possess protective effects against liver injury caused by acetaminophen and ethanol in mice [18,19] and may contribute to stress and anti-inflammatory response. Thus, the present study indicates that GLY-treated female fish are more susceptible to oxidative stress and ROS effects.

Hypotaurine, a sulfinic acid intermediate in taurine biosynthesis, is another metabolite possessing antioxidant capacity and acting as an osmolyte inside the cells [20,21]. The liver of GLY-exposed females contained an increased level of hypotaurine within the taurine and hypotaurine metabolism pathway. This is consistent with the reported increase level of taurine in *Carassius auratus* exposed to a GLY-based herbicide [15]. Furthermore, hepatic increase of this metabolite was also found in diet-induced rats with non-alcoholic fatty liver disease (NAFLD), a pathologic condition fostering inflammation and production of oxidative stress [22]. Studies concerning humans with acute liver failure demonstrated increased hypotaurine and decreased ATP together with lower levels of intermediates involved in glycolysis and pentose phosphate pathways [23]. The latter study indicates that, in case of liver abnormality, a hepatic shift from glycolysis to trans-sulfuration metabolism may occur. Similar conditions appear to be present in the liver of GLY- exposed female fish displaying a decrease in purine and pyrimidine intermediates and His levels which are derived from PRPP and the pentose phosphate pathway, together with increased hypotaurine levels. Furthermore, in GLY-exposed female fish, sucrose levels increased in connection with the starch, sucrose, and galactose metabolic pathways, suggesting a contaminant-induced reduction in the metabolism of sugars. Similar results were reported in goldfish treated with Nongteshi^®^ [15], leading to decreased pyruvate kinase activity, a rate-limiting step glycolytic enzyme.

Exposure to GLY resulted in increased DHA in female fish, potentially impacting fatty acid metabolism. There is evidence that DHA reduces inflammatory and oxidative stress response [24] and liver fibrosis [25] in rats treated with the organophosphate chlorpyrifos or carbon tetrachloride, respectively. The enhancement of DHA and *cat* production in the liver of females exposed to GLY could be a possible mechanism to counteract GLY-induced hepatotoxicity and inflammation mediated by *lifaf*, *cxcl8b.1* and interleukins.

Exposure of females to GLY also increased transcript levels of the genes involved in the stress response, including *nr3c1* and *hsp70.2*, possibly mediated by glucocorticoid receptors [26,27,28]. The observed increase in the level of *hsp70.2,* which codes for HSP70, a heat shock protein chaperon of relevance in the fish cellular response to stress [29], provides further evidence that female zebrafish were responding to GLY-induced stress. In this context, *hsp70.2* expression is a reliable biomarker of xenobiotic exposure since it is upregulated by a different broad spectrum of pollutants [30,31,32,33,34]. Surprisingly, the GLY-exposed male metabolic phenotype was not significantly different from CTRL in the multivariate analysis. In this regard, only a decrease in aminoadipic acid levels in connection with the lysine degradation pathway was found to be significant. Aminoadipic acid is metabolized into glutaryl-CoA, which is then converted to acetyl-CoA and enters the citrate cycle. A possible explanation for the observed decrease of this metabolite is its enhanced catabolism to form intermediates used in energy production.

Decreased transcript levels of genes involved in oxidative stress response, including *sod1*, *sod2,* and *gpx1a* in GLY-exposed male fish, indicate a diminished capacity to counteract ROS-induced stress. The GLY-induced oxidative stress response was previously reported in rats [3,35] and fish [6,36]. In the male liver, the expression of stress response genes showed an increased level of the glucocorticoid receptor with decreased *hsp70.2* level indicating that GLY exposure also affects the stress response, but in contrast to females, male fish may not be able to counteract the stress response using a similar mechanism to females. Our results are consistent with a previous study in *Oreochromis niloticus* chronically exposed to GLY [6].

There is clear evidence that GLY acts as an endocrine disruptor compound (EDC) [37]. This compound interacts with nuclear hormone receptors, including the estrogen receptor alpha (ERα). In this regard, GLY exposure enhanced ERα activation and stimulated its transcriptional activation in human breast cancer cells [37]. Furthermore, in vivo studies demonstrated the ability of GLY to alter hypothalamic gene expression, increase testosterone synthesis in the ovaries, and alter estradiol-sensitive genes in rats [37]. It is interesting that estradiol stimulation was observed to enhance amidophosphoribosyltransferase (PPAT) gene expression in breast cancer cells, which are involved in PRPP conversion to 5-phosphoribosyl-1-amine (PRA) for purine de novo synthesis [38]. By altering steroid hormone levels, it is possible to speculate that the decreased levels of purine intermediates are due to an impairment of this conversion through altered hormone homeostasis. Therefore, a possible explanation of the sex-specific effects observed in our study could be the capacity of GLY to interact with ER. In females, ERs are key receptors that maintain ovarian granulosa cell differentiation and follicle and oocyte growth and development [39]. However, beyond the canonical role in reproductive functions, estrogens also play a role in the regulation of other physiological functions such as immune response, growth, neuronal function, and metabolism [40,41,42]. Thus, by targeting estrogen receptors which are more abundant in females than in males, GLY could have different sex-related disruptive effects.

Overall, the present results provide evidence that chronic exposure to GLY dysregulates metabolism in zebrafish. We provide evidence that GLY exposure leads to stress response in *Danio rerio* in a sex-dependent manner. Our study also highlights the acute toxicity of GLY when formulated with coadjutants and surfactants such as commonly used GLY-based herbicides. Thus, it would be hard to suggest a safe environmental level for GLY as its toxicity changes significantly when combined with coadjutants. Further studies are needed to provide more evidence on the toxicity of GLY combined with coadjutants to better assess the risks associated with this type of herbicide.

## 4. Materials and Methods

### 4.1. Animal Treatment and Maintenance

In order to assess GLY toxicity, 90 adult zebrafish (*D. rerio*, AB wild-type strain) were divided into four aquaria of 20 L each (20 fish/tank), in duplicate, under controlled conditions (28.0 ± 0.5 °C) and with a 14/10 h light/dark cycle in oxygenated water. Fish were fed twice a day with commercial food (TetraMin Granules; Tetra, Melle, Germany) in the morning and *Artemia salina* in the afternoon, with a quantity of food ranging from 2.5% to 3.0% of fish body weight. Water was changed every two days. Two experimental groups were set up as follow: 

Control (CTRL): fish fed twice a day with commercial food (TetraMin Granules; Tetra, Melle, Germany) and *Artemia salina.*

Glyphosate (GLY): fish were fed commercial food and *Artemia salina*, and were exposed to 700 µg/L of glyphosate (98% analytical purity, Sigma-Aldrich, Milano, Italy) via water (GLY powder was dissolved in water before administration every two days.

All groups were sampled after 4 weeks of treatment. Fish were euthanized with 500 mg/L MS-222 (3-aminobenzoic acid ethyl ester, Sigma Aldrich) buffered to pH 7.4. For metabolomic analysis livers were pooled (three livers per pool), while for transcript analysis five livers were sampled. All samples were stored at −80 °C until processed for analysis. Procedures involving animals were conducted following the University of Calgary animal care protocol (AC16-0160) for care and use of experimental animals, all efforts were made to minimize suffering.

### 4.2. Metabolite Extraction and UHPLC-ESI-MS Analysis

Liver metabolite extraction, separation, and identification were performed on seven pools of female livers and four pools of male livers for each investigated group, as well as the quality control group (QC; *n* = 4) generated by pooling random samples as described previously [43]. The number of pools analyzed was determined on the basis of the fish sex ratio while sampling. Effort was made to analyze the same number of pools between CTRL and GLY females or males. Liver samples (20 mg) were homogenized using a bead-beating homogenizer (TissueLyser II, QIAGEN) and extracted with methanol (ratio of 1:20 sample (mg): methanol (μL)). After 20 min of centrifugation at 13,500 rpm, the supernatant was collected and stored at −80 °C until further analysis. The quality control group (QC) was generated by pooling 20 mg from five random samples, extracted following the same protocol and analyzed in four statistical replicates. Metabolites were analyzed using ultra-high-performance liquid chromatography (UHPLC) mass spectrometry (MS), with a hydrophilic-interaction liquid chromatography column (Syncronis HILIC, Thermo Fisher, stationary phase) to separate the metabolites. High-resolution full-scan MS data were acquired on a Thermo Fisher Scientific Q-Exactive HF mass spectrometer using negative-mode electrospray ionization. MAVEN freeware and an m/z and retention time standard were used for targeted profiling of the MS spectra and identification of the metabolites. Peak intensity was measured as area top, representing the average intensity of the top three points of the peak [43].

### 4.3. RNA Extraction and cDNA Synthesis

Extraction of total RNA was performed from five female and five male livers for CTRL and GLY experimental groups, using the RNAeasy^®^ Minikit (Qiagen, Milano, Italy) following the manufacturer’s instructions, and eluted in RNase-free water at a final volume of 50 µL. RNA quality evaluation and cDNA synthesis were assessed as previously described [44] and are here briefly reported. RNA concentration was determined using a NanophotometerTMP-Class (Implen GmbH, Munich, Germany), and 28S and 18S ribosomal RNA was stained using ethidium bromide on 1% agarose gel to determine RNA integrity.

DNase treatment (10 IU at 37 °C for 10 min, MBI Fermentas, Milano, Italy) was performed on total RNA to digest genomic DNA, and 1 µg RNA was used for cDNA synthesis performed using the iScript cDNA Synthesis Kit (Bio-Rad, Milano, Italy) according to the manufacturer’s instructions.

### 4.4. Real-Time PCR

In order to perform qRT-PCRs, a CFX thermal cycler was used with SYBR green, as described in [44], and ribosomal protein 13 (*rpl13*) and ribosomal protein 0 (*rplp0*) mRNAs were used as internal standards in each sample to standardize the results and eliminate variation in mRNA and cDNA quantity and quality. Table 4 reports primer sequences, GenBank accession numbers, and primer efficiency of the examined genes.

Furthermore, the calculation of mRNA levels of target genes analyzed was performed using the geometric mean of the two reference genes after demonstrating that they were stably expressed by the geNorm algorithm, both applications implemented in the Bio-Rad CFX Manager 3.1. software. Gene transcript expression alterations among experimental groups are reported as relative mRNA abundance (arbitrary units). Primers were used at a final concentration of 10 pmol/mL.

### 4.5. Statistical Analysis

#### 4.5.1. Metabolomic Statistical Analysis

Normalization, transformation, and scaling (normalization by a median, log transformation, and pareto scaling) were conducted prior to conducting multivariate analysis. SIMCA software (Umetrics, Umeå, Sweden), was used for partial least squares-discriminant analysis (PLS-DA) and hierarchical analysis model generation. Quality assessment of the models was indicated by R2 and Q2 > 0.5, and models with *p* < 0.05 were considered significant [43]. The MetaboAnalyst 5.0 online platform (University of Aberta, Alberta Canada) was used to perform unsupervised principal component analysis (PCA) on all treatments and QC to identify potential outliers and assess the LC-MS reliability. QC samples are usually some random samples that are pooled and extracted together. This extract is then aliquoted in different wells of the 96-well plate and analyzed before and after the samples of interest as technical replicates, to confirm the reliability of the technique.

Hierarchical cluster analysis was performed in a “bottom-up” manner using the Ward clustering algorithm. VIP (variable importance in projection) score > 1 was used as a threshold for the identification of significant metabolites in each PLS-DA model. VIP > 1 was used for hierarchical clustering (Ward clustering, Euclidean distance), univariate analysis t-test, and pathway analysis using Metaboanalyst 5.0 as described before [43]. A significant threshold of *p*-value adjusted using false discovery rate (FDR) < 0.05 was used to assess statistically significant differences among experimental groups.

#### 4.5.2. Pathway Analysis

Metabolites with VIP score > 1 in each OPLS-DA model, were used to perform pathway analysis (MetPA) using the Metaboanalyst 5.0 platform [43]. This technique considers the concentration of each metabolite, using quantitative enrichment analysis (QEA) and the position in the pathway, using topological analysis. For these two parameters, global test and relative-betweenness centrality algorithms were selected, respectively, using the KEGG pathway library of zebrafish (*Danio rerio*) as reference.

#### 4.5.3. Gene Expression Statistical Analysis

T-test analysis was performed to analyze transcript expression. Statistical software package Prism5 (GraphPad Software, Inc., San Diego, CA, USA) was used for statistical analyses with *p* < 0.05 as threshold for statistical significance.

## Figures and Tables

**Table 1:** VIP score of metabolites found with PLS-DA analysis.

(**a**)		(**b**)	
**Metabolites**	**VIP (12)**	**Metabolites**	**VIP (25)**
N-Acetyl-L-methionine	1.3711	Hippuric acid	1.98682
Docosahexaenoic acid	1.29572	Aminoadipic acid	1.86273
Sucrose	1.26731	Docosahexaenoic acid	1.68523
L-Homoserine	1.25124	N-Acetylleucine	1.66798
Azelaic acid	1.23396	Cytidine monophosphate	1.65299
Hypotaurine	1.23293	Uracil	1.64123
Adenosine monophosphate	1.13408	N-Acetyl-L-alanine	1.63765
Uridine 5′-monophosphate	1.07566	Uridine 5′-monophosphate	1.61569
Guanosine monophosphate	1.038	Hypoxanthine	1.58986
Uric acid	1.02329	Sucrose	1.57968
L-Histidine	1.02007	Acetoacetic acid	1.57219
Inosinic acid	1.00379	L-Alanine	1.44502
		Betaine	1.32999
		Pyrrolidonecarboxylic acid	1.29793
		N-Acetyl-L-methionine	1.21579
		Pyridoxal	1.15716
		Azelaic acid	1.11747
		Uridine diphosphate-N-acetylglucosamine	1.09991
		L-Asparagine	1.06611
		Inosinic acid	1.04028
		Ophthalmic acid	1.03219
		Guanosine monophosphate	1.03092
		O-Phosphoethanolamine	1.01278
		L-Homoserine	1.01177
		Fumaric acid	1.00205

VIPs > 1 found in PLS-DA-built models of CTRL vs. GLY comparison in (a) females and (b) males.

**Table 2:** Metabolites modulated by GLY exposure in female and male livers.

(**a**)				(**b**)			
**Metabolite Name**	***p*** **-Value**	**FDR**		**Metabolite Name**	***p*** **-Value**	**FDR**	
Sucrose	0.001231	0.008438	↑	Aminoadipic acid	0.000236	0.0017	↓
Azelaic acid	0.001406	0.008438	↓				
Hypotaurine	0.002545	0.01018	↑				
L-Homoserine	0.005126	0.012943	↑				
Uric acid	0.005954	0.012943	↑				
Adenosine monophosphate	0.006472	0.012943	↓				
Uridine 5′-monophosphate	0.010393	0.015703	↓				
L-Histidine	0.010824	0.015703	↓				
Docosahexaenoic acid	0.011778	0.015703	↑				
Guanosine monophosphate	0.018083	0.019942	↓				
Inosinic acid	0.01828	0.019942	↓				

Multiple *t*-test analysis results showing metabolites significantly upregulated (**↑**) or down-regulated (**↓**) in CTRL vs. GLY comparison in (a) females and (b) males. *p*-value and FDR were reported; FDR < 0.05 was considered significant.

**Table 3:** Summary of the main altered metabolic pathways in fish liver exposed to GLY.

(**a**)			(**b**)		
**Pathway Name**	**FDR**	**Impact**	**Pathway Name**	**FDR**	**Impact**
Galactose metabolism	0.005414	0.04029	Lysine degradation	0.0051841	0
Starch and sucrose metabolism	0.005414	0.01534			
Taurine and hypotaurine metabolism	0.0076346	0.4			
Purine metabolism	0.011778	0.23384			
Pyrimidine metabolism	0.011778	0.09066			
Histidine metabolism	0.011778	0.2381			
Nitrogen metabolism	0.011778	0			
Aminoacyl-tRNA biosynthesis	0.011778	0			
Biosynthesis of unsaturated fatty acids	0.011778	0			

MetPA table showing main altered metabolic pathways found in CTRL vs. GLY comparison in (a) female and (b) male livers. FDR values < 0.05 were considered statistically significantly altered and are shown. Impact is a value which refers to altered metabolite centrality in the changed pathways found.

**Table 4:** Primer list.

Name	Forword	Reverse	Efficiency (Tm°)	Accession Number
*cat*	CCAAGGTCTGGTCCCATAAA	GCACATGGGTCCATCTCTC	60°	NM_130912
*sod1*	GTCGTCTGGCTTGTGGAGTG	TGTCAGCGGGCTAGTGCTT	60°	NM_131294
*sod2*	CCGGACTATGTTAAGGCCATCT	ACACTCGGTTGCTCTCTTTTCTCT	60°	NM_199976
*gpx1a*	ACCTGTCCGCGAAACTATTG	TGACTGTTGTGCCTCAAAG	59°	NM_001007281.2
*nr3c1*	CGCCTTTAATCATGGGAGAA	AGACCTTGGTCCCCTTCACT	58°	NM_001020711.3
*hsp70.2*	TGTTCAGTTCTCTGCCGTTG	AAAGCACTGAGGGACGCTAA	58°	NM_001362360.1
*litaf*	TTGTGGTGGGGTTTGATG	TTGGGGCATTTTATTTTGTAAG	53°	NM_001002184.1
*il 1b*	GTGGATTGGGGTTTGATGTG	GCTGGGGATGTGGACTTC	54°	NM_212844.2
*cxcl8b.1*	ACTCGGACTGAAGGTGACTC	CCACGTCTCGGTAGGATTGAG	58°	NM_001327985
*il 10*	GCCGTGGAGCAGGTGAAG	GAAGATGTCAAACTCACTCATGGCT	58°	NM_001020785
*esr1*	GGTCCAGTGTGGTGTCCTCT	AGAAAGCTTTGCATCCCTCA	58°	NM_152959.1
*rpl13a*	TCTGGAGACTGTAAGAGGTATGC	AGACGCACAATCTTGAGAGCAG	59°	NM_198143
*rplp0*	CTGAACATCTCGCCCTTCTC	TAGCCGATCTGCAGACACAC	60°	NM_131580

## Data Availability

Not applicable.
